# Impact of intercropping grass on the soil rhizosphere microbial community and soil ecosystem function in a walnut orchard

**DOI:** 10.3389/fmicb.2023.1137590

**Published:** 2023-03-14

**Authors:** Changxi Wang, Qiang Liang, Jianning Liu, Rui Zhou, Xinya Lang, Shengyi Xu, Xichen Li, Andi Gong, Yutian Mu, Hongcheng Fang, Ke Qiang Yang

**Affiliations:** ^1^College of Forestry, Shandong Agricultural University, Tai'an, Shandong, China; ^2^State Forestry and Grassland Administration Key Laboratory of Silviculture in the Downstream Areas of the Yellow River, Shandong Taishan Forest Ecosystem Research Station, Tai'an, Shandong, China

**Keywords:** walnut, grass intercropping, rhizosphere microorganism, bacterial community, nitrogen cycle, carbohydrate metabolism

## Abstract

The intercropping of grass in orchards has beneficial effects on soil properties and soil microbial communities and is an important soil management measure for improving orchard productivity and land-use efficiency. However, few studies have explored the effects of grass intercropping on rhizosphere microorganisms in walnut orchards. In this study, we explored the microbial communities of clear tillage (CT), walnut/ryegrass (*Lolium perenne* L.) (Lp), and walnut/hairy vetch (*Vicia villosa* Roth.) (Vv) intercropping system using MiSeq sequencing and metagenomic sequencing. The results revealed that the composition and structure of the soil bacterial community changed significantly with walnut/Vv intercropping compared to CT and walnut/Lp intercropping. Moreover, the walnut/hairy vetch intercropping system had the most complex connections between bacterial taxa. In addition, we found that the soil microorganisms of walnut/Vv intercropping had a higher potential for nitrogen cycling and carbohydrate metabolism, which may be related to the functions of *Burkholderia, Rhodopseudomonas, Pseudomonas, Agrobacterium, Paraburkholderia*, and *Flavobacterium*. Overall, this study provided a theoretical basis for understanding the microbial communities associated with grass intercropping in walnut orchards, providing better guidance for the management of walnut orchards.

## Introduction

Walnut (*Juglans regia* L.) is an important woody oil tree species that is widely cultivated globally because it is rich in proteins, unsaturated fatty acids, vitamins, and minerals conducive to health (Bernard et al., 2018; Ji et al., 2021). However, with the expansion of the cultivation area and extensive management measures, the yield of high-quality walnut nuts results in poor economic benefits. In recent years, intercropping with grass in orchards has emerged as a simple and efficient measure for enhancing fruit tree cultivation and management (Li et al., 2022). Therefore, studying grass intercropping in walnut orchards and clarifying the knowledge gap related to the rhizosphere microbiome under this intercropping system is crucial for the development of the walnut industry. Grass cultivation stabilizes soil aggregates, conserves water and soil, improves soil physical properties, and increases soil nutrient availability, which are beneficial for tree growth and development (Hoagland et al., 2008; Ramos et al., 2011; Wei et al., 2017). In addition, grass cultivation can also improve the community composition of soil microorganisms, thus changing the interaction between plants and microorganisms (Rieux et al., 1999).

Soil microorganisms, encompassing bacteria, fungi, and archaea, play a significant role in conserving multiple soil ecosystem functions, including soil nutrient cycling and soil organic matter content and composition (Wagg et al., 2014; Sun et al., 2015; Fierer, 2017). The pH, organic matter, nutrient content, and particle fraction of the soil are shown to impact microbial community diversity and components (Aciego Pietri and Brookes, 2009; Cheng et al., 2021; Zheng et al., 2021). Correspondingly, functional microbial biomass also promotes soil nitrification progress and soil enzyme activities (Shi et al., 2017; Chen et al., 2021). Recently, the critical role of soil microorganisms in plant growth has also been widely studied. *Bacillus* strains isolated from the rhizosphere soil of sugarcane have an N-fixation function and can resist *Sporisorium scitamineum* and *Ceratocystis paradoxa* (Singh et al., 2020). Furthermore, soil microbiomes can regulate plant growth, drought stress, and the leaf metabolome of *Arabidopsis thaliana* (Badri et al., 2013; Zolla et al., 2013). Therefore, understanding the effects of orchard grass on soil microorganism functions is necessary to accurately assess ecosystem functions.

Recently, with the advancement of high-throughput sequencing and next-generation DNA sequencing (NGS) approaches, the composition and structure of soil microbial communities have been extensively studied (Navarrete et al., 2015). Intercropping of Trifolium (*Trifolium repens* L.)/cucumber and mustard (*Brassica juncea* L.)/cucumber notably increases soil bacterial and fungal community diversity based on MiSeq sequencing of 16S rRNA and ITS genes (Li and Wu, 2018). Illumina MiSeq high-throughput sequencing technology indicated that continuous sugarcane (*Saccharum officinarum*) cropping reduces the diversity of bacterial and fungal communities and the soil microbial community structure (Pang et al., 2021). In addition, the emergence of metagenomics has provided deep insights into the function of soil microbial communities. Metagenomics and comparative genomics have demonstrated that carbohydrate and secondary metabolite transport functionalities are overrepresented within drought-enriched taxa (Xu et al., 2021). The functions of secondary metabolite biosynthesis genes in soil bacterial communities are also characterized using metagenomics (Crits-Christoph et al., 2018).

In this study, we performed MiSeq sequencing of 16S rRNA genes and metagenomic sequencing of rhizosphere bacterial communities to analyze their differences in clear tillage (CT), ryegrass (*Lolium perenne* L.)/walnut (Lp), and hairy vetch (*Vicia villosa* Roth.)/walnut (Vv) intercropping systems. The results showed that the soil properties and microbial structure and diversity were considerably affected by grass intercropping. Furthermore, the soil microorganisms of walnut/Vv intercropping had a higher potential for nitrogen cycling and carbohydrate metabolism, which may be related to the functions of *Burkholderia, Rhodopseudomonas, Pseudomonas, Agrobacterium, Paraburkholderia*, and *Flavobacterium* as they were significantly and positively correlated with soil organic matter (SOM) and total nitrogen (TN). These results will provide metagenomic insights for evaluating the effects of walnut/grass intercropping on the microecological environment.

## Materials and methods

### Site description and sample collection

The study was conducted in a walnut orchard at the Forestry Experimental Station of Shandong Agricultural University in Tai'an city, Shandong Province, China (117°8′59″E, 36°10′16″N), at an altitude of 135.6 m, with an average annual rainfall of 740 mm, and an average annual temperature of 12.9°C. The soil type is brown soil.

The walnut tree variety was “Ruixiu,” which had been cultivated at the site for 8 years. The trial consisted of three treatments, with perennial ryegrass and hairy vetch as intercropping grasses and a clear tillage control. Ryegrass/walnut, hairy vetch/walnut intercropping systems, and clear tillage were represented by Lp, Vv, and CT, respectively. The perennial ryegrass and hairy vetch were planted for 3 consecutive years (during which the weed-free area was maintained), and the clear tillage plots were managed with normal weeding for 3 consecutive years. Three plots were set up for each treatment, with a total of nine plots separated by a barrier strip between the different treatments. Five soil samples were collected and mixed from each plot. In mid-June 2021, the fine roots of the walnut trees were collected from the same part of the walnut tree roots (20 cm from the surface) in each sample plot. The surface soil was removed by shaking the roots, and the rhizosphere soil was collected into sterile bags placed in 4°C ice boxes, which were brought back to the laboratory immediately. The fresh soil samples were divided into two parts: One part was dried, ground, and sieved through a 100-mesh sieve for soil nutrient and enzyme activity determination, and the other part was stored at −80°C for the determination of various microbial indicators.

### Analysis of soil physicochemical properties and enzyme activity

Soil pH was determined by an acidity meter (soil/water = 1/2.5) according to Pang et al. (2021). The SOM was assessed by the potassium dichromate volumetric-external heating method, TN was determined using the Kjeldahl method (Ding et al., 2019), and total phosphorus (TP) and available phosphorus (AP) were determined by the Mo-Sb colorimetric method, total potassium (TK) and available potassium (AK) was determined by the atomic absorption method (Li and Liu, 2017), and available nitrogen (AN) was determined by the alkaline hydrolyzable diffusion method (Liu et al., 2009).

Urease (UE), sucrase (SC), neutral phosphatase (NP), dehydrogenase (DH), and nitrate reductase (NR) were determined by the visible spectrophotometric method, and the kits were purchased from Suzhou Grace Biotechnology Co., Ltd.

### DNA extraction and PCR amplification of 16S rRNA

A Mo Bio PowerSoil^®^ DNA kit (QIAGEN, Shanghai, China) was used to extract the total soil microbial DNA (genomic DNA, gDNA) according to the kit instructions, and the quality and concentration of the extracted DNA were determined by 0.8% agarose gel electrophoresis and a NanoDrop 2000 spectrophotometer, respectively (Thermo Fisher Scientific, Waltham, MA, USA).

The highly variable V3–V4 region of the bacterial 16S rRNA gene was amplified using the 341F (CCTAYGGGRBGCASCAG) and 806R (GGACTACNNGGGTATC TAAT) primers. The PCR was performed in a total volume of 50 μl, which contained 25 μl of PCR Mix Buffer (using Phusion High-Fidelity PCR Master Mix with HF Buffer), 3 μl of DMSO, 3 μl each of F/R primers, and 10 ng of gDNA. The amplification conditions were as follows: one pre-denaturation cycle at 98°C for 30 s, 30 denaturation cycles at 98°C for 15 s, annealing at 58°C for 15 s, extension at 72°C for 15 s, and a final extension at 72°C for 1 min. The PCR amplification products were detected by 2% agarose gel electrophoresis, and the target fragments were recovered by a QIAquick Gel Extraction Kit (QIAGEN, Valencia, CA, USA, cat: 28706X4). The recovered PCR products were quantified by a Microplate reader (BioTek, FLx800) using a Quant-iT PicoGreen dsDNA Assay Kit (Thermo Fisher Scientific, Carlsbad, CA, USA, Cat# P7589) with reference to the preliminary quantification results of electrophoresis. Equal amounts of PCR products were used to construct sequencing libraries using TruSeq Nano DNA LT Library Prep Kit (Illumina, San Diego, CA, USA), and the constructed libraries were sequenced by the Illumina MiSeq platform (Illumina, San Diego, CA, USA) with a sequencing strategy of PE250 (paired-end sequencing 250 × 2).

The sequenced data were analyzed using the Quantitative Insights Into Microbial Ecology (QIIME2) pipeline. In brief, DADA2 was used to initially process the reads by removing the PCR primer sequences, splice sequences, and sequences of low quality (*Q* < 30). Then, the naive Bayesian classifier method in the DADA2 pipeline (v 1.16.0) algorithm was used to cluster the reads into amplicon sequencing variants (ASVs), and the Silva reference database (version 138.1) was used to assign the taxonomy of each ASV. QIIME 2 was then applied to calculate alpha diversity, including Chao1 richness, Pielou's evenness, and Shannon diversity. Principal coordinate analysis (PCoA) was performed to assess beta diversity, and the ANOSIM test was used to examine the significance using the “vegan” (v 2.6-2) package (Oksanen et al., 2014).

### Metagenomic sequencing analysis

The same soil DNA extractions were used for shotgun metagenomic sequencing. In brief, a library with an insert size of 300 bp was constructed using ~1 μg of DNA and then sequenced by the Illumina HiSeq 4000 platform using the PE150 strategy (paired-end sequencing 150 × 2). The raw downstream data were quality-controlled using Trimmomatic (v 0.39) to remove reads with average quality scores of <30 (Q30) and length of <50 bp. The clean reads after QC were assembled using Megahit (v1.2.9), and contigs with lengths >300 bp were retained. The open reading frames (ORFs) in the contigs were predicted using Megahit (v1.2.9) with default parameters, and ORFs with lengths >100 bp were clustered as the non-redundant gene catalog using CD-HIT (v4.8.1) with 95% identity and 90% coverage. Metabolic function annotations of non-redundant genes were obtained using KofamScan (v1.3.0) for homology comparison with the Kyoto Encyclopedia of Genes and Genomes (KEGG) database. The expression of genes in different samples was calculated using SOAP2 (v2.21) as the reads per kilobase of reads per million (RPKM). Taxonomic classification of bacteria, archaea, fungi, and viruses was assigned to metagenomic reads using Kraken2, an improved metagenomic taxonomy classifier that utilizes k-mer-based algorithms. Differences at the species level were analyzed using the “edgeR (v3.38.1)” package, and the screening criteria for differences were log2 (FC) > 1||log2 (FC) <−1 and *p*-value <0.005. Differentiated species were plotted using the “ggplot2” (v3.4.0) package, and enriched or depleted species belonging to phyla were visualized using the “fmsb” (0.7.3) package.

### Rhizosphere microbiota network construction

To reduce the complexity of the network, species with abundance >0.1%, which accounted for more than 85% of the total samples on average, were selected for Spearman's correlation calculations (*P* < 0.05), and the network was visualized using Gephi.

### Statistical analysis

The redundancy analysis (RDA) function of the vegan (v2.6-2) package in R was used to conduct RDA of multiple correlation variations among environmental factors and community composition at the genus level (permutations = 999). LDA effect size (LEfSe) analysis based on genus level among the groups was combined with non-parametric Kruskal–Wallis and Wilcoxon's rank sum tests to screen species with significant differences between groups and conduct a comparative analysis of differences. Then, the “psych” (v2.2.5) package was used to calculate Spearman's correlations and significance between soil physicochemical properties and differential genus abundance, and the correlation was demonstrated using the “pheatmap” (v1.0.12) package.

One-way analyses of variance (ANOVA) with Tukey's HSD multiple range tests were performed to detect differences in soil physicochemical properties and alpha diversity indices between different groups using SPSS 19.0 (IBM Corp., Armonk, NY, USA). PCoA based on the Bray–Curtis distance matrix was conducted with the vegan package to ordinate the microbial community structure between different treatments. Kraken2 was used for species annotation, and then Bracken was used to calculate the relative abundance of the species annotation results. KofamScan was used for KEGG annotations, and eggNOG-mapper software was used to annotate the Clusters of Orthologous Genes (COG) and Carbohydrate-Active enZYmes (CAZy) results.

## Results

### The physicochemical properties and enzyme activity of the soil from different sampling sites

The soil physicochemical properties of the walnut orchards among the three sampling sites, CT, and grassed with Lp and Vv are shown in Table 1. The Lp and Vv treatments both significantly increased the SOM and AK content compared to the CT treatment, whereas the pH decreased significantly. The content of SOM and AK increased by 15.09 and 44.57%, respectively, in Lp and by 53.03 and 79.13%, respectively, in Vv, compared to that in the CT. The Vv treatment significantly increased the TN and AP content compared to the control, but the content of TN and AP was not significantly different between the Lp treatment and the CT treatment. In addition, the activities of soil urease (S-UE), soil sucrase (S-SC), soil neutral phosphatase (S-NP), and soil dehydrogenase (SDHA) in the two intercropping systems were significantly higher than those of the CT. These results showed that intercropping with grass can improve the soil physicochemical properties in walnut orchards, particularly the Vv intercropping treatment.

**Table 1:** Basic physicochemical parameters and five major enzyme activities among the treatments.

**Parameter**	**CT**	**Lp**	**Vv**
pH	7.737 ± 0.13^a^	7.430 ± 0.04^b^	7.440 ± 0.04^b^
SOM	9.877 ± 0.40^c^	11.367 ± 0.48^b^	15.115 ± 0.37^a^
TN	1.060 ± 0.04^b^	1.203 ± 0.04^b^	1.577 ± 0.11^a^
TP	0.593 ± 0.01^a^	0.592 ± 0.01^a^	0.593 ± 0.01^a^
TK	24.537 ± 0.40^a^	20.400 ± 0.34^b^	22.247 ± 1.97^ab^
AN	78.633 ± 5.25^a^	76.300 ± 5.56^a^	78.633 ± 1.76^a^
AP	22.935 ± 4.95^b^	34.974 ± 4.85^ab^	48.798 ± 12.09^a^
AK	30.667 ± 4.04^c^	44.333 ± 1.62^b^	54.933 ± 0.92^a^
S-UE	0.138 ± 0.01^c^	0.197 ± 0.01^b^	0.266 ± 0.01^a^
S-SC	17.479 ± 0.85^c^	22.373 ± 0.22^b^	31.062 ± 0.80^a^
S-NP	0.609 ± 0.02^c^	1.121 ± 0.04^b^	1.366 ± 0.12^a^
SDHA	5,047.443 ± 204.71^c^	5,774.403 ± 125.39^b^	6,555.893 ± 192.83^a^
S-NR	12.116 ± 0.64^a^	7.348 ± 0.90^b^	8.395 ± 1.07^b^

Values are the means (*n* = 3) ± the standard deviation of the mean, and different letters indicate significant differences at *P* < 0.05. pH, potential of hydrogen; SOM, soil organic matter (g·kg^−1^); TN, TP, and TK indicate total nitrogen, total phosphorus, and total potassium, respectively (g·kg^−1^); AN, AP, and AK indicate available nitrogen, available phosphorus, and available potassium, respectively (mg·kg^−1^); S-UE, soil urease (mg·g^−1^); S-SC, soil sucrase (mg·g^−1^); S-NP, soil neutral phosphatase (mg·g^−1^); SDHA, soil dehydrogenase (μg·g^−1^); S-NR, soil nitrate reductase (μg·g^−1^). CT, clear tillage; Lp, *Lolium perenne* L.; Vv, *Vicia villosa* Roth.

### Characteristics of the different grassed soil bacterial communities

**Figure 1:**
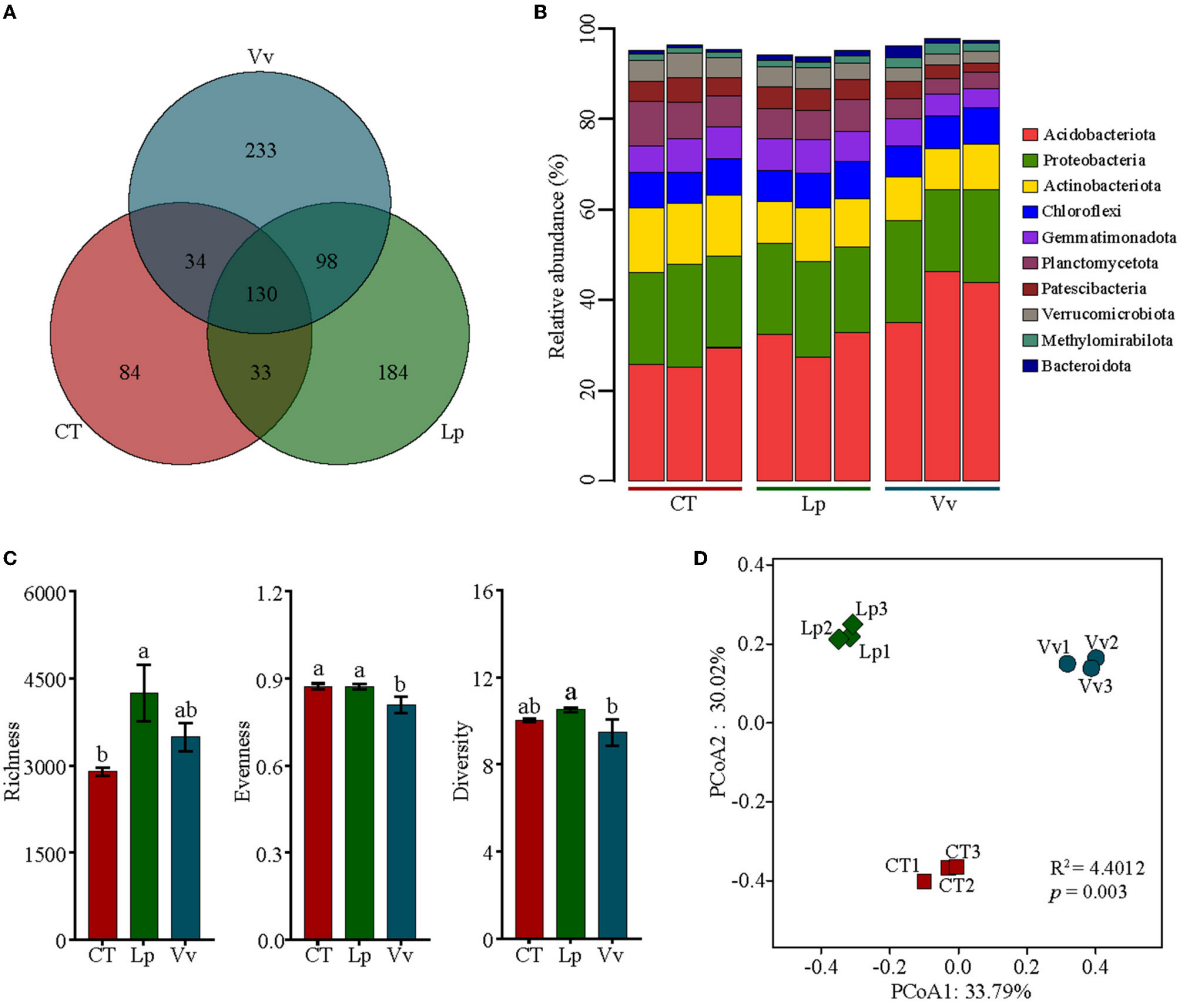
Structure of soil bacterial communities based on 16S rRNA gene sequencing in three soil samples. **(A)** Venn diagrams of exclusive and shared bacterial species. **(B)** Relative abundances of the top-10 dominant strains at the phylum level. **(C)** Alpha diversity estimates of bacterial communities. **(D)** Principal coordinate analysis (PCoA) plot of ASVs based on the Bray–Curtis distance matrix.

To estimate the effects of orchard grass on soil bacterial communities, we initially undertook 16S rRNA gene sequencing. A total of 951,611 sequences clustered into 11,925 ASVs across all nine soil samples after quality filtering. For the bacterial community, 50 phyla, 132 classes, 342 orders, 512 families, 868 genera, and 796 species were detected from three soil samples, of which 281, 445, and 495 species were detected in CT, Lp, and Vv, respectively (Figure 1A, Supplementary Table 1). Among them, even if all samples had the most dominant phyla, namely Acidobacteria, Proteobacteria, and Actinobacteria, their relative abundances varied among the different treatments (Figure 1B). For example, the relative abundance of Acidobacteria in the soils vegetated with Vv was 41.7%, which was significantly higher than that in Lp (30.8%) and CT soil (26.8%) (Supplementary Table 2). To further clarify the soil bacterial community changes induced by cropping with various types of grass, we identified the significantly enriched/depleted ASVs in Lp vs. CT and Vv vs. CT based on the edgeR analysis. A total of 24 ASVs and 55 ASVs were significantly enriched in Lp vs. CT and Vv vs. CT, respectively. These ASVs were mainly distributed across two phyla: Firmicutes and Bacteroidetes. Meanwhile, there were a total of 94 ASVs and 58 ASVs depleted in Lp vs. CT and Vv vs. CT, respectively, which were also mainly distributed across Firmicutes and Bacteroidetes (Supplementary Figure 1).

Then, we estimated the alpha diversity indices (Chao richness, Pielou's evenness, and Shannon index) based on the ASV level. Orchard grass significantly increased bacterial ASV richness compared to the control soil as shown in Figure 1C. The bacterial ASV richness indices were 4,241 and 3,492 in the soil vegetated with Lp and Vv, respectively, which were higher than that in the control soil (2,893). The evenness and diversity level of the soil with Vv were slightly lower, while no difference was observed between Lp and CT (Figure 1C). Meanwhile, PCoA of CT, Lp, and Vv was performed to identify the dissimilarity between the treatments. The PCoA showed that PC1 explained 33.79% of the variation and PC2 explained 30.02% of the variation, and the bacteria in the soils vegetated with Lp and Vv were distinctly separated from those in CT (*R*^2^ = 4.4012, *p* = 0.003) (Figure 1D). These results revealed that the intercropping grass in the walnut orchard significantly changed the soil bacterial community composition and structure, and the distinction was even more significant for the soil vegetated with Vv.

### Characteristics of the different soil microbiomes using metagenomics

**Figure 2:**
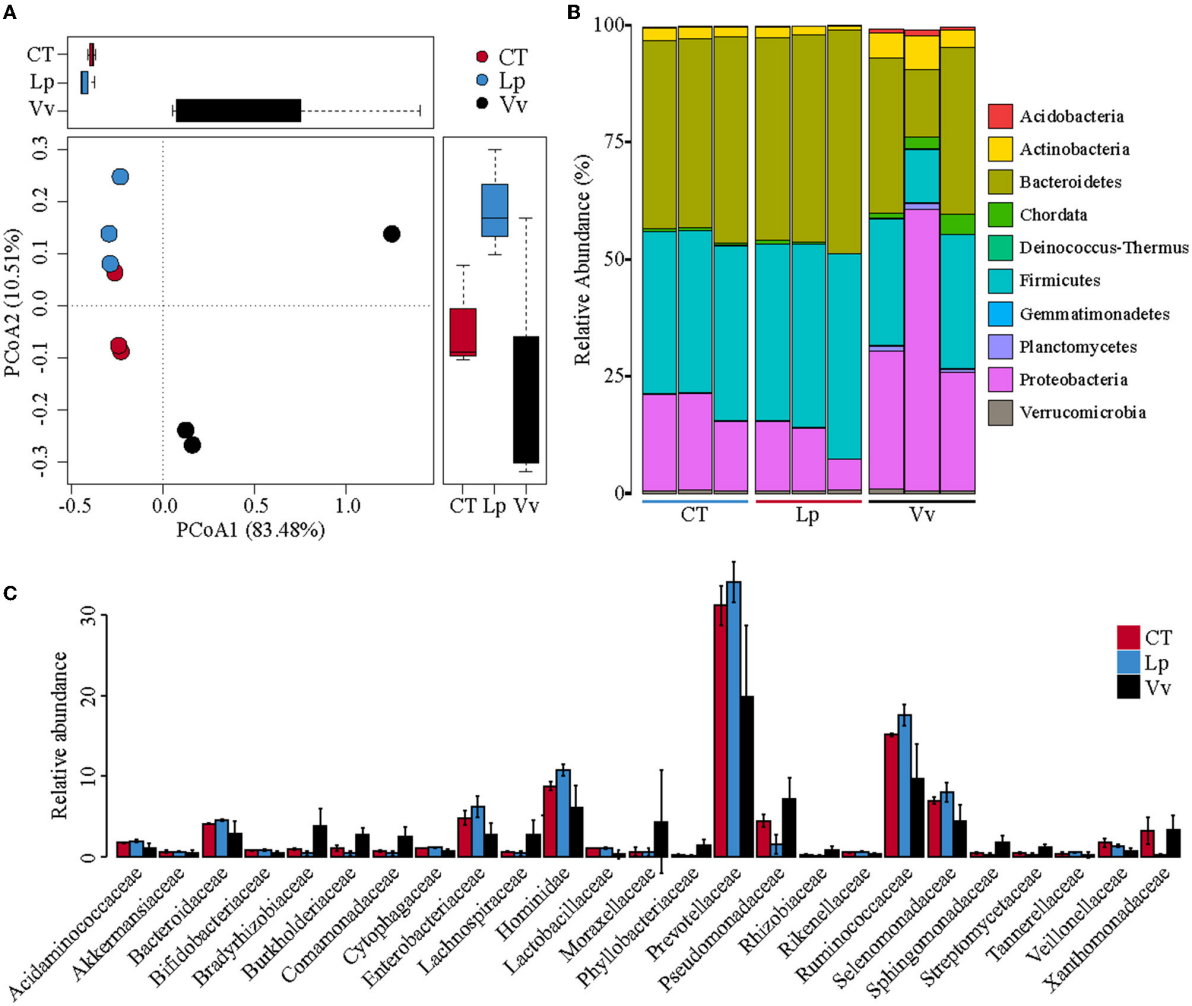
Microbiome compositions of bacterial taxa based on metagenomic sequencing between different treatments. **(A)** Principal coordinate analysis (PCoA) of different soil samples based on the Bray–Curtis distance matrix at the species level. **(B)** Relative abundances of different phyla in the soil samples. **(C)** Taxonomic histogram of the top 25 most-abundant bacteria at the family level.

We further confirmed that the intercropping with grass in the walnut orchard induced changes in the soil microbial community based on metagenomic sequencing. The PCoA showed that walnut/Vv was clearly clustered into one group and was significantly separated from the CT and walnut/Lp soils along the first principal coordinate (83.48%) using Bray–Curtis dissimilarity (Figure 2A). The variation in the PCoA of the metagenomic sequences was lower than that of 16S, which may be caused by the different compositions of the bacterial populations according to the two sequencing methods. Consistent with the 16S rRNA sequencing analysis, at the phylum level, soils vegetated with Vv were characterized by a significant increase in Acidobacteria, Actinobacteria, and Proteobacteria and a decrease in Bacteroidetes compared to the control, while the composition and structure of the soil microbiome in soils vegetated with Lp did not differ significantly from that in CT (Figure 2B). At the family level, an expansion of Bradyrhizobiaceae, Burkholderiaceae, Comamonadaceae, and Moraxellaceae was observed in walnut/Vv soils compared to CT and Lp soils, while several families exhibited a significantly lower relative abundance, including Enterobacteriaceae, Hominidae, Prevotellaceae, Ruminococcaceae, and Selenomonadaceae (Figure 2C).

**Figure 3:**
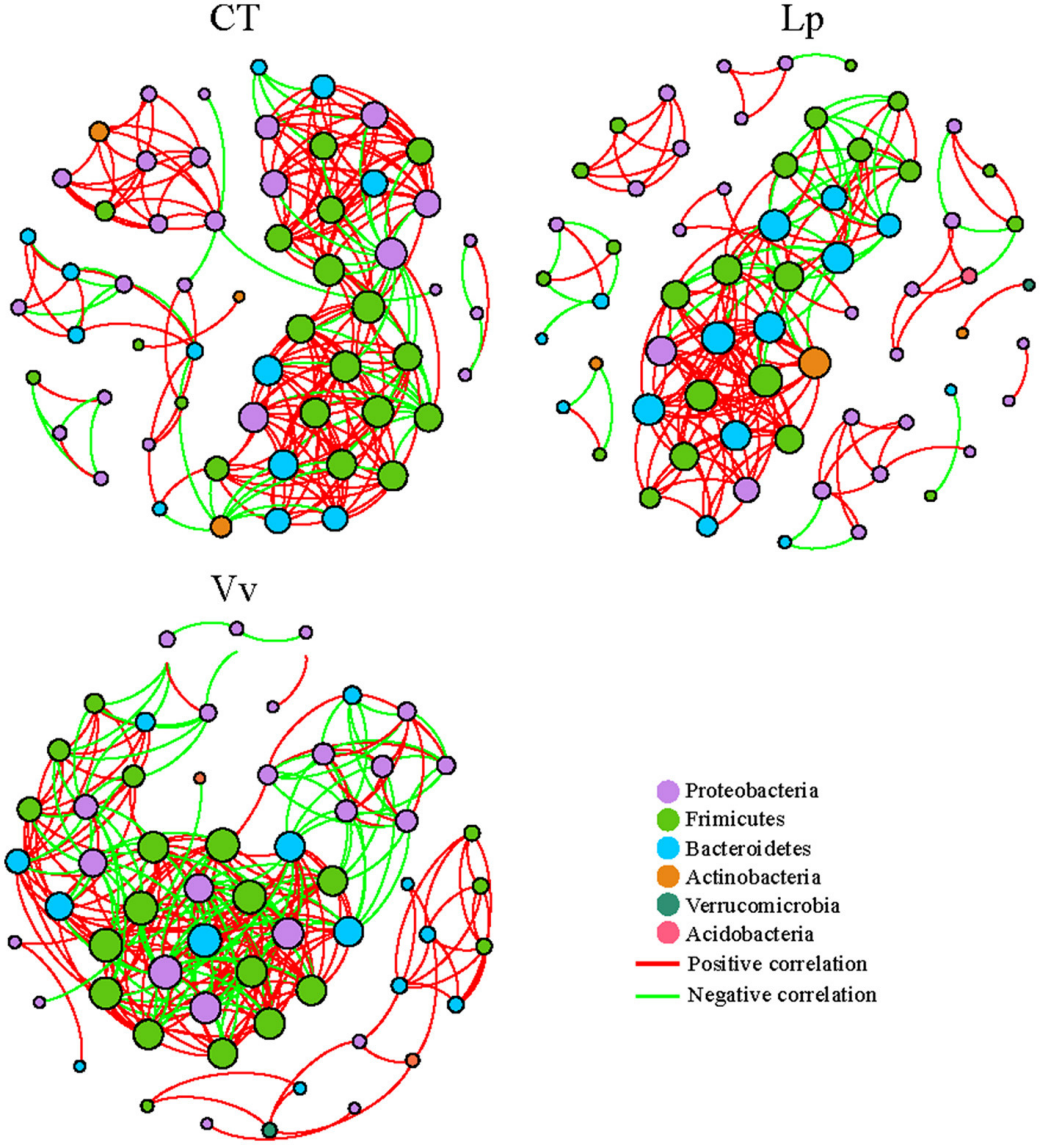
Variation of rhizospheric bacterial networks caused by intercropping. Co-occurrence networks of the different treatments. Only the nodes with Spearman's correlation coefficient *r* > 0.8, *P* < 0.01 or *r* < −0.8, *P* < 0.01 are shown. Nodes represent the species identified by Kraken2 in meta, and the edges between the nodes indicate significant correlations. A red edge indicates positive covariation between two individual nodes, while a green edge indicates negative covariation. The size of each node is proportional to the number of connections, while the colors of the nodes indicate major phyla.

Meanwhile, to determine the differences in rhizosphere bacterial assemblages in the different intercropping systems, we constructed co-occurrence networks. Our results showed that the bacterial assemblages of Vv formed a larger network diameter, more edges, larger average path length, and higher average degree compared to CT and Lp. The ratio of positive to negative connections was 1.4 in Vv and 3.6 and 2.9 in CT and Lp, respectively, indicating the presence of more competitive or inhibitory connections in Vv (Figure 3, Supplementary Table 3). Overall, the walnut/Vv intercropping system had the most complex rhizosphere microbial network.

### Functional characteristics of the different soil microbiomes

**Figure 4:**
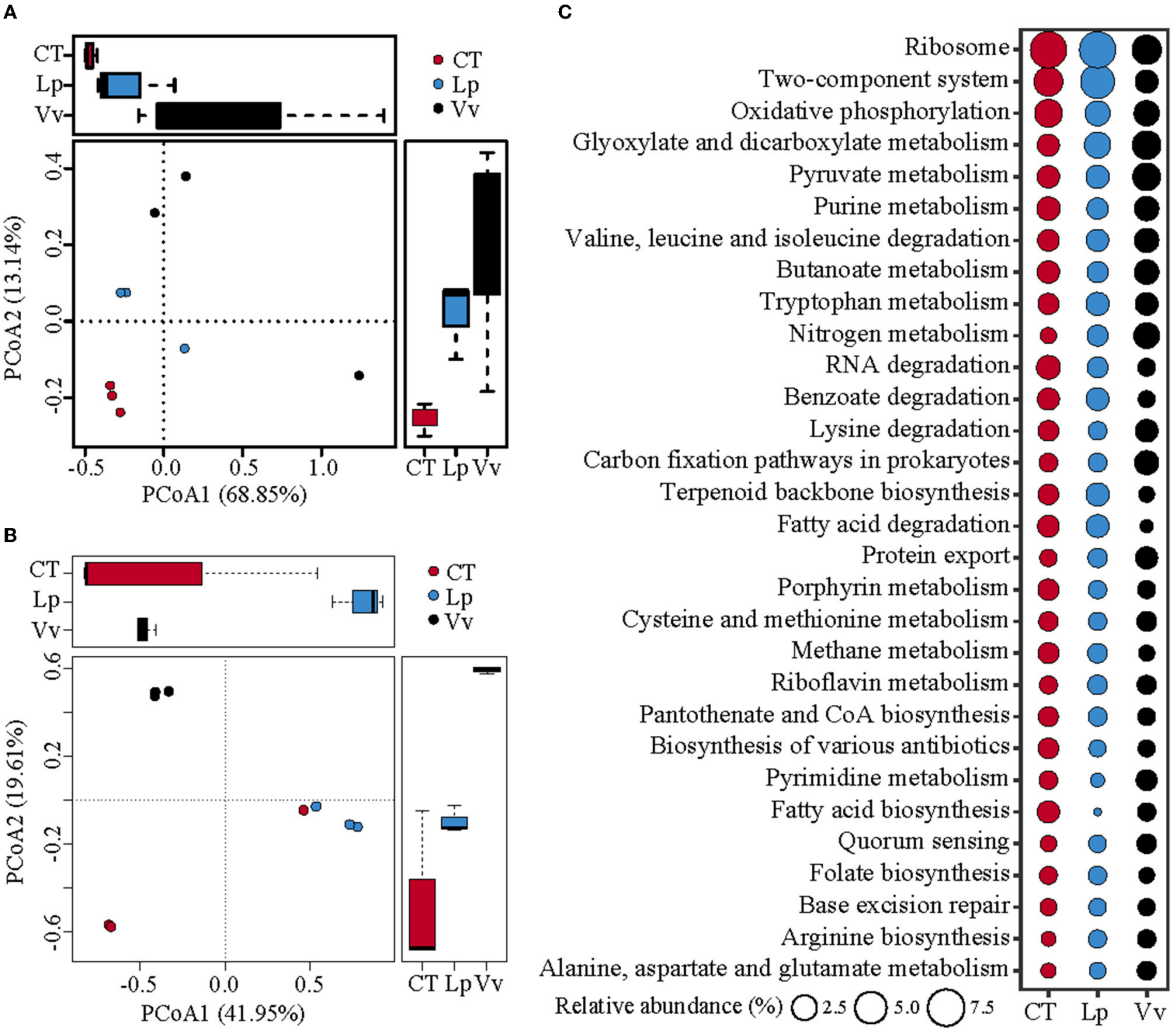
Overview of functional genes in the soil under different treatments. **(A)** Principal coordinate analysis (PCoA) plot of KEGG functions on the basis of Bray–Curtis dissimilarity. **(B)** PCoA plot of COG functions on the basis of Bray–Curtis dissimilarity. **(C)** Relative abundance of the 30 most-abundant KEGG pathways under different treatments.

To explore the characteristics of the potential functional genes in the different vegetated soils, functional annotation of the assembled metagenomes was performed using the KEGG and EggNOG databases. The PCoA results of the KEGG pathway showed that along the first principal coordinate, the Vv treatment showed maximal differences (68.85%) compared to the CT treatment, followed by the Lp treatment (Figure 4A). The PCoA results of EggNOG exhibited the same trend, and the maximum difference between Vv and CT was 41.95% (Figure 4B). Compared to the CT and Lp treatments, the microbiomes in Vv exhibited a higher metabolic potential for substance and energy metabolism (Figure 4C, Supplementary Figure 2). For instance, the relative abundances of carbohydrate metabolism genes were 17.77 in the soils vegetated with Vv, which was significantly higher than the values in the soils vegetated with Lp (10.95%) and the control (10.64%). The relative abundance ofenergy metabolism in the soils vegetated with Vv was 12.44%, while that in the Lp and CT treatments was 9.70% and 9.99, respectively (Supplementary Figure 2, Table 4). Similarly, the EggNOG annotation also revealed that the soil of Vv exhibited significantly increased carbohydrate transport and metabolism, energy production and conversion, and amino acid transport and metabolism (Supplementary Figure 3).

**Figure 5:**
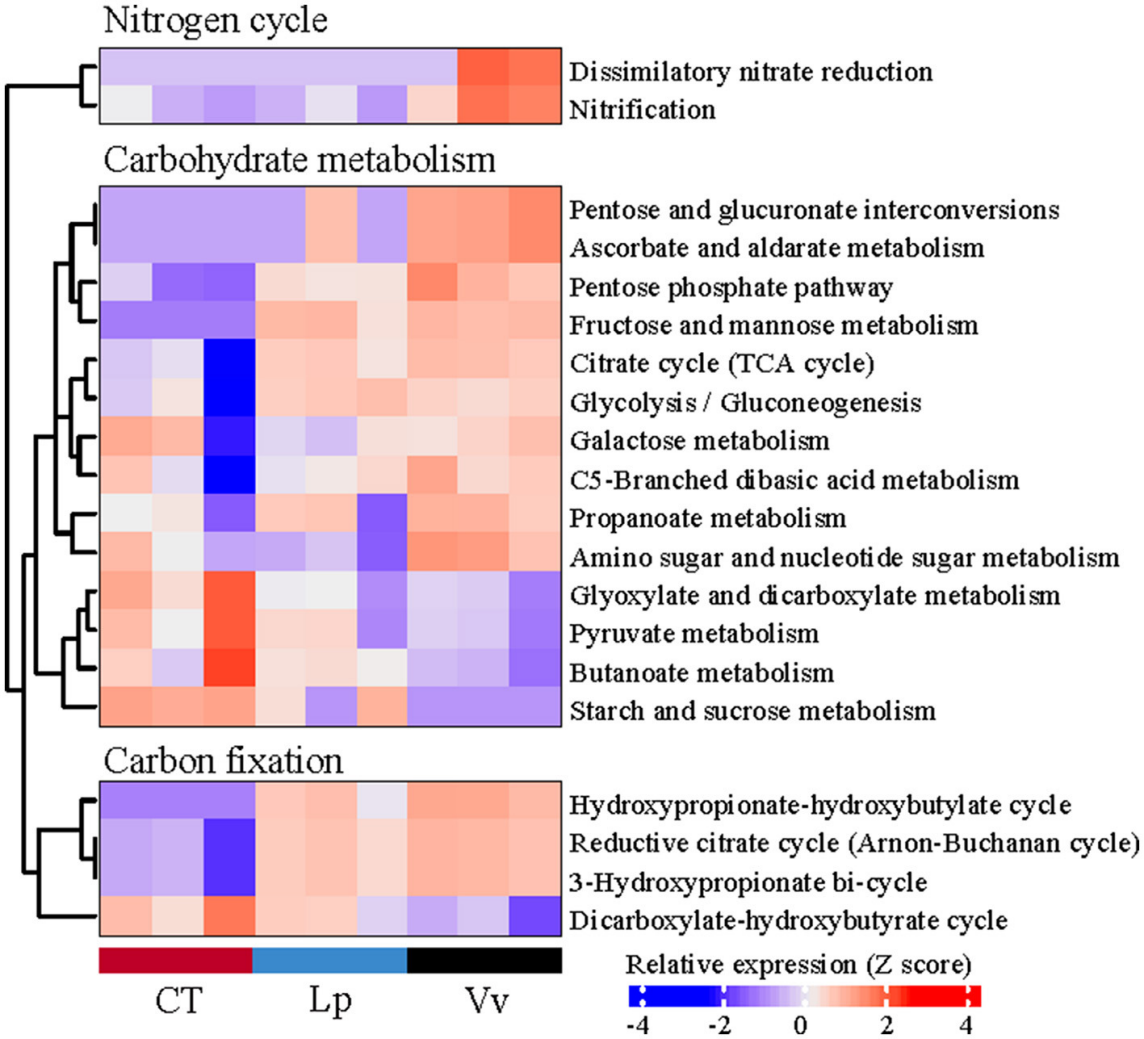
Heatmaps showing differences in the relative abundances of functions related to the *N* cycle, C metabolism, and C fixation in all treatments.

Meanwhile, the LEfSe analysis of the KEGG pathways showed that the microbiomes in the Vv soils exhibited a higher metabolic potential for N and C (Figure 4C). In the N cycle, Vv increased the functional potential of dissimilatory nitrate reduction and nitrification compared with the CT and Lp treatments. In addition, Vv also increased the metabolic potential of functions related to carbohydrate metabolism and carbon fixation. Among them, pentose and glucuronate interconversions, ascorbate and aldarate metabolism, and amino sugar and nucleotide sugar metabolism in carbohydrate metabolism were significantly enriched in the Vv treatment. In addition, the hydroxypropionate–hydroxybutyrate cycle, reductive citrate cycle, and 3-hydroxypropionate bi-cycle in carbon fixation were also significantly enriched in the Vv treatment (Figure 5).

### Relationship between soil properties and microbial structure

**Figure 6:**
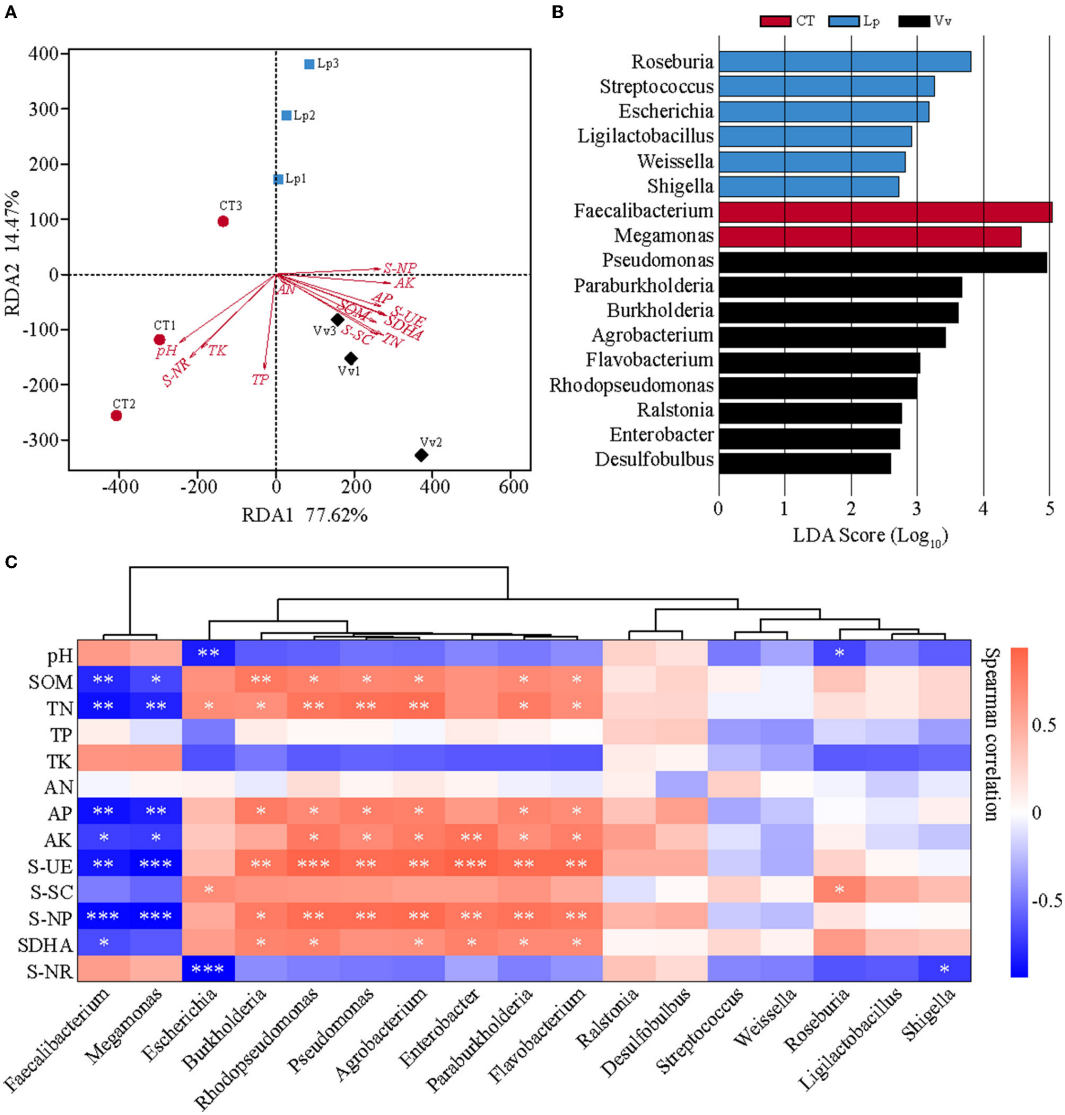
Relationship between soil properties and microbial structure. **(A)** Redundancy analysis (RDA) of soil physicochemical properties. **(B)** LDA effect size (LEfSe) analysis of all bacteria at the genus level. **(C)** Spearman's correlation analysis between environmental factors and the dominant genera of the bacterial communities in different soils.

To define the effects of the changes in soil physicochemical properties on the soil microbial community compositions and structure, RDA was conducted. It showed that the first two RDA components (RDA1 and RDA2) explained 77.62 and 14.47% of the total variance in the bacterial community, respectively. The AK, AP, TN, SOM, S-UE, SDHA, and S-SC were significantly correlated with bacterial communities in the Vv soil samples, whereas only PH, S-NR, and TK were correlated with the bacterial communities in the CT soil samples (Figure 6A, Supplementary Table 5). Meanwhile, we conducted LEfSe analysis of all bacteria at the genus level to determine specific bacteria with significant differences in abundance in different soils based on LDA > 2.5. *Faecalibacterium* and *Megamonas* were enriched in the CT soil samples. *Roseburia, Streptococcus, Escherichia, Ligilactobacillus, Weissella*, and *Shigella* were enriched in the Lp soil samples. *Pseudomonas, Paraburkholderia, Burkholderia, Agrobacterium, Flavobacterium, Rhodopseudomonas, Ralstonia, Enterobacter*, and *Desulfobulbus* were significantly enriched in the Vv soil samples (Figure 6B, Supplementary Table 6).

Then, Spearman's correlation analysis was conducted to define the correlation between environmental factors and the dominant genera of the bacterial communities in different soils. The bacterial genera were identified as significantly correlated with the soil properties based on the threshold of |*R*| > 0.70 and *P* < 0.05. Among the bacterial genera, *Faecalibacterium, Megamonas, Escherichia, Burkholderia, Rhodopseudomonas, Pseudomonas, Agrobacterium, Enterobacter, Paraburkholderia*, and *Flavobacterium* were the key taxa highly related to soil properties. For instance, *Burkholderia* was significantly and positively correlated with SOM (*R* = 0.80, *P* < 0.05), TN (*R* = 0.67, *P* < 0.05), AP (*R* = 0.78, *P* < 0.05), S-UE (*R* = 0.82, *P* < 0.05), S-NP (*R* = 0.78, *P* < 0.05), and SDHA (*R* = 0.73, *P* < 0.05). By contrast, *Faecalibacterium* was negatively correlated with SOM (*R* = −0.80, *P* < 0.05), TN (*R* = −0.88, *P* < 0.05), AP (*R* = −0.88, *P* < 0.05), AK (*R* = −0.71, *P* < 0.05), S-UE (*R* = −0.87, *P* < 0.05), and S-NP (*R* = −0.92, *P* < 0.05). Among them, *Pseudomonas, Paraburkholderia, Burkholderia, Agrobacterium, Flavobacterium*, and *Rhodopseudomonas*, which were enriched in the Vv soil samples, were significantly and positively correlated with TN (Figure 6C, Supplementary Table 7).

## Discussion

Intercropping with grass in orchards is considered a simple and efficient management measure to improve SOM and nutrient contents (Wu et al., 2011; Qiao et al., 2014). The plants reported for orchard intercropping are generally natural grass, ryegrass, alfalfa, clover, and *Vulpia myuros* (Coller et al., 2019; Sun et al., 2022). A previous study found that interplanting with *Vulpia myuros* could enhance the physicochemical properties of soils and provide nutrients for fruit trees (Brown and Rice, 2010). In this study, the soil physicochemical properties were analyzed in CT, walnut/Lp, and walnut/Vv intercropping systems. Compared to the CT treatment, the Lp and Vv treatments both significantly increased the content of SOM and AK and the activities of S-UE, S-SC, S-NP, and SDHA. Moreover, the Vv treatment significantly increased the content of TN and AP compared to the control, which was not significantly different between the Lp treatment and the CT treatment.

Intercropping with grass also modulated the abundance and composition of the soil microbial community. In apple orchards, intercropping with licorice and grass could significantly affect the composition and structure of the soil microbial community (Li et al., 2022). Intercropping with white clover or alfalfa dramatically enhanced the microbial biomass carbon content and the abundance of Actinomycetes, and intercropping with ryegrass significantly increased the abundance of *Nitrospira* species (Rodríguez-Loinaz et al., 2008). Moreover, Bever found that legume intercropping plants not only improved the variety of the microbial community but also increased the abundance of beneficial soil microbes in the orchard (Bever, 2015). The results of this study provide further evidence that intercropping with grass can alter the soil bacterial community composition and structure through 16S gene sequencing and metagenomic sequencing. Interestingly, the composition and structure of the bacterial community in the soil intercropped with Vv were more significantly changed than those in CT and intercropping with Lp. In addition, the co-occurrence networks confirmed that the walnut/Vv intercropping system had the most complex rhizosphere microbial network. This result may be due to the rapid creeping growth of Vv in spring, which then decays after withering in summer to improve SOM and allow *Metarhizium anisopliae* and *Bacillus subtilis* to thrive.

As a leguminous plant, Vv alters the nutrient status of soils by fixing atmospheric N in association with compatible rhizobia, mainly *Rhizobium leguminosarum* symbiovar *viceae* (Gentry et al., 2013; Yuan et al., 2016; Renzi et al., 2020). The exudates of leguminous plant roots modify the soil microbial diversity, structure, and functional groups associated with the *N* cycle (Zhang et al., 2021). Recent studies have reported that leguminous plants promote biological *N* fixation to enhance soil *N* and *C* storage (Sun et al., 2022). Chinese fir plantations intercropped with leguminous plants can promote the diversity of soil microbial communities by increasing the soil N content (Zhang et al., 2020). Similar to the aforementioned research, compared to CT and Lp, the TN content was significantly increased in the Vv treatment. In addition, the microbiomes in the Vv soils exhibited a higher metabolic potential for substance and energy metabolism, especially the *N* cycle and *C* metabolism based on the LEfSe analysis of KEGG pathways. These results indicate that intercropping with Vv in a walnut orchard significantly increased the *C* and *N* content through the soil bacterial community enrichment in *C* metabolism and the *N* cycle.

Previous studies have shown that soil physicochemical properties play a key role in mediating microbial community structure and many bacterial communities arehighly correlated with specific soil factors, which have been used as indicators of soil condition (Lauber et al., 2008; Kuramae et al., 2012; Val-Moraes et al., 2016). Pang et al. (2021) found that Arthrobacter abundance showed a positive correlation with pH and that *Bradyrhizobium, Sphingomonas, Streptomyces*, and *Burkholderia* showed a negative correlation with pH and TS in continuous cropping of sugarcane. In our study, the RDA showed that AK, AP, TN, SOM, S-UE, SDHA, and S-SC were significantly correlated with the bacterial communities in the Vv soil samples. In addition, walnut orchard intercropping with Vv better enriched the bacterial genera including *Pseudomonas, Paraburkholderia, Burkholderia, Agrobacterium, Flavobacterium, Rhodopseudomonas, Ralstonia, Enterobacter*, and *Desulfobulbus*. Among them, *Pseudomonas, Paraburkholderia, Burkholderia, Agrobacterium, Flavobacterium*, and *Rhodopseudomonas* were significantly and positively correlated with TN. These results indicated that these bacterial genera likely play an important role in the *N* cycle, but the specific functions need further verification.

## Conclusion

In summary, both the Lp and Vv intercropping systems altered the soil physicochemical properties and microbial community structure compared to CT, with the latter being particularly significant. The KEGG pathway results showed that the soil microorganisms of walnut/Vv intercropping had a higher potential for *N* cycling and *C* metabolism. Meanwhile, we hypothesized that TN-related bacterial genera such as *Burkholderia, Rhodopseudomonas, Pseudomonas, Agrobacterium, Paraburkholderia*, and *Flavobacterium* may play an important role in the *N* cycle. Therefore, our future research focus is to isolate these novel bacterial genera and verify their interactions with host plants. Overall, these findings can suggest agricultural management and microbial applications in walnut orchards.

## Data availability statement

The datasets presented in this study can be found in online repositories. The names of the repository/repositories and accession number(s) can be found in the article/Supplementary material.

## Author contributions

HF and KQY designed and supervised the research. CW performed the experiments. JL and QL analyzed the data. CW and HF wrote the original draft manuscript. RZ, XLa, SX, XLi, AG, and YM reviewed and edited the manuscript. All authors reviewed and approved the manuscript.

## Supplementary material

Supplementary Figure 1 Different types of grasses are enriched and depleted for certain metagenomic species in the soil. The enriched/depleted species are the species with significantly different abundance compared to the species in CT samples based on “edgeR” analysis. **(A)** Enrichment and depletion of the species included in the soils vegetated with different grasses. **(B)** Phylum annotation of the differentially enriched or depleted species.

Supplementary Figure 2 Relative abundance of KEGG functional pathways.

Supplementary Figure 3 Distribution of all EggNOG genes under different treatments, grouped into EggNOG categories.

Supplementary Table 1 Soil bacterial communities in different treatments.

Supplementary Table 2 Relative abundances of the top-10 dominant strains at the phylum level.

Supplementary Table 3 Topological properties of the co-occurrence networks.

Supplementary Table 4 Relative abundance of KEGG functional pathways.

Supplementary Table 5 Redundancy analysis (RDA) of soil physicochemical properties.

Supplementary Table 6 LDA effect size (LEfSe) analysis of all bacteria at the genus level.

Supplementary Table 7 Spearman's correlation analysis between environmental factors and the dominant genera of the bacterial communities in different soils.
