# Supplementary figures and images for: Impact of intercropping grass on the soil rhizosphere microbial community and soil ecosystem function in a walnut orchard
Source: Front Microbiol. 2023 Mar 14;14:1137590. doi: 10.3389/fmicb.2023.1137590 (PMC10046309; doi:10.3389/fmicb.2023.1137590)

A

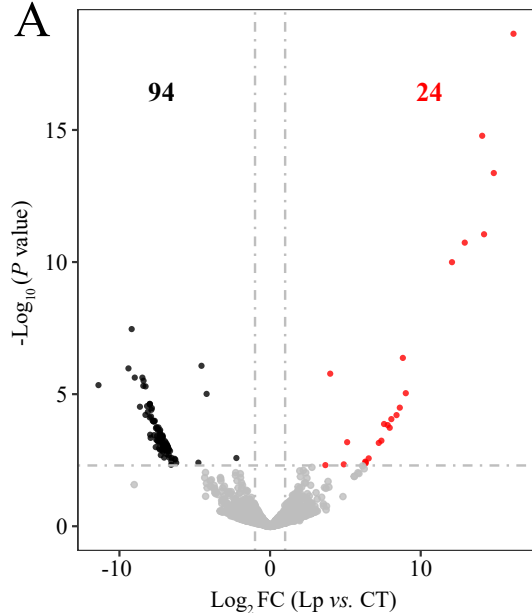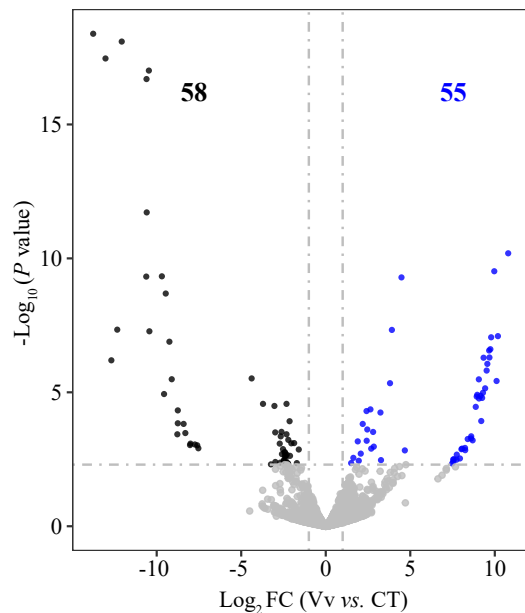

B

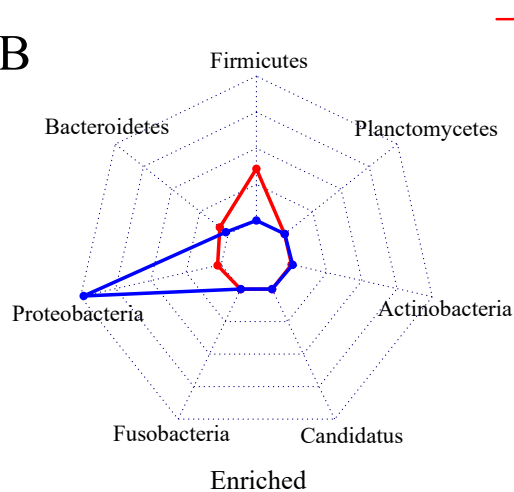

— Lp — Vv

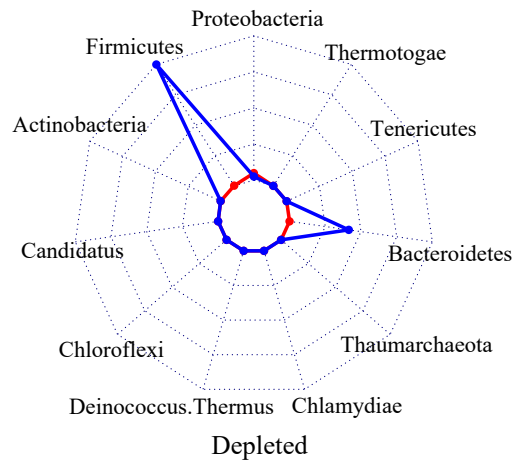

Supplement: Supplementary Figure 1 — Different types of grasses are enriched and depleted for certain metagenomic species in the soil. The enriched/depleted species are the species with significantly different abundance compared to the species in CT samples based on “edgeR” analysis. (A) Enrichment and depletion of the species included in the soils vegetated with different grasses. (B) Phylum annotation of the differentially enriched or depleted species. [file Data_Sheet_1.PDF]

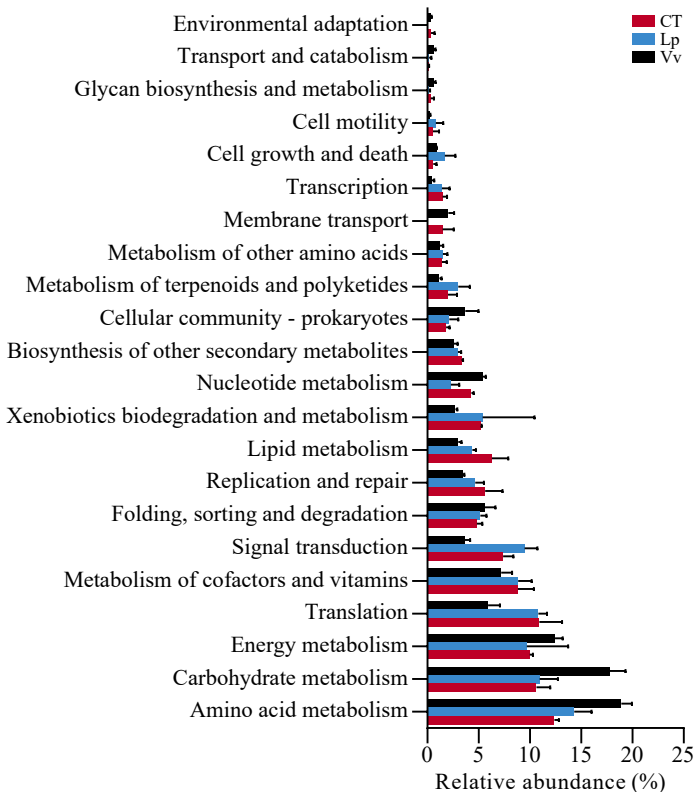

Supplement: Supplementary Figure 2 — Relative abundance of KEGG functional pathways. [file Data_Sheet_2.PDF]

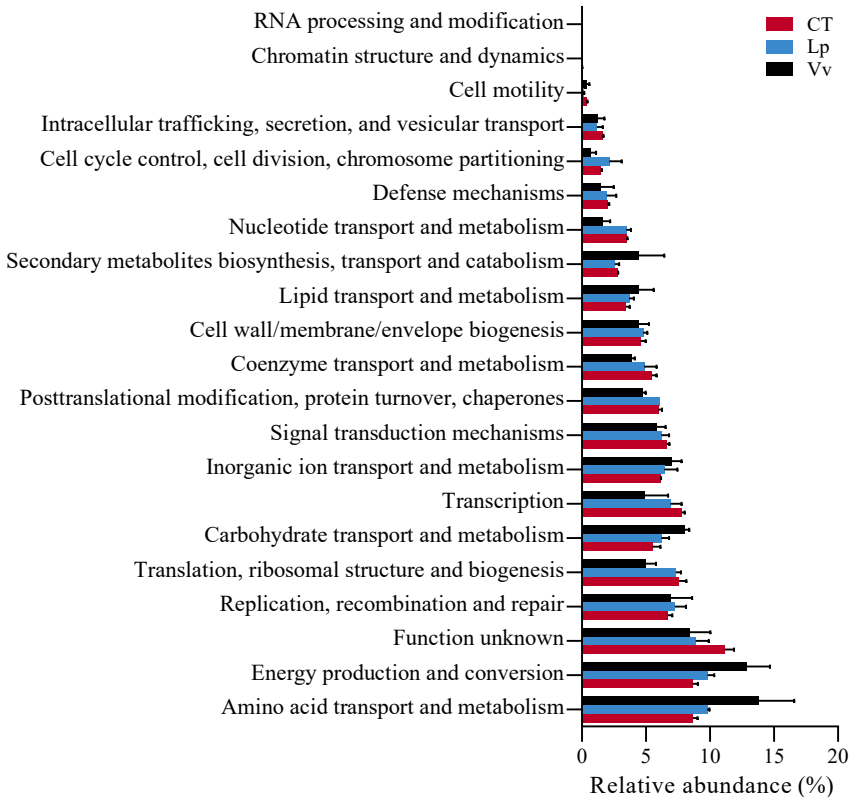

Supplement: Supplementary Figure 3 — Distribution of all EggNOG genes under different treatments, grouped into EggNOG categories. [file Data_Sheet_3.PDF]
